# Integrating customer relationship management with business intelligence platform design: a design science approach

**DOI:** 10.1007/s44362-026-00024-x

**Published:** 2026-09-15

**Authors:** Min Zhang, Byron Graham, Julie McCandless, Louise Skeath

**Affiliations:** 1 Queen’s University Belfast, Belfast, UK; 2 SDG, Armagh, UK

**Keywords:** Business intelligence platform, Data analytics, Customer relationship management, Knowledge transfer, Small and medium-sized enterprise

## Abstract

Situated within the broader business analytics paradigm, business intelligence (BI) is important in providing small and medium-sized enterprises (SMEs) with the information needed to manage customer relationships successfully. However, SMEs face specific challenges when integrating customer relationship management (CRM) with the development of BI platforms. Drawing on the design science research methodology, this study combines CRM and BI to design, develop, implement, and evaluate a CRM-BI platform alongside strategic and process artefacts within an SME that supplies specialist construction materials. First, we design the customer strategy and the approach to developing customer relationships. We then design a customer experience map and service blueprint, which inform the customer engagement model. Drawing on these strategic and operational CRM artefacts, a BI dashboard is developed to support a broader review of business practices and facilitate data-driven decision-making. Successful implementation of the CRM-BI platform enabled the SME to transition from reactive decision-making to proactive, data-driven strategic decision-making, identify new business opportunities, and improve overall business performance. The platform also supported a programme of strategic change, restructuring, improved accountability, and innovation. The study contributes to theory and practice through novel artefacts, design principles and design knowledge.

**Supplementary Information:**

The online version contains supplementary material available at https://doi.org/10.1007/s44362-026-00024-x.

## Introduction

Customer relationship management (CRM) focuses on the use of market intelligence to develop and maintain profitable customer relationships (Payne et al., 2005; Zablah et al., 2004). It is recognised that technology is an important part of CRM, alongside the business processes and strategy needed to build successful customer relationships (Zablah et al., 2004). Business analytics has been identified as playing an important role in CRM (Nam et al., 2019), with applications ranging from data warehousing and storage solutions (Watson, 2002), business intelligence and data mining (Phan & Vogel, 2010), text analytics (Xu et al., 2017), through to advanced artificial intelligence (Ledro et al., 2023).

Firms can use business analytics to enhance performance and operational efficiency (Gupta & George, 2016), improve decision-making effectiveness (Li et al., 2022), increase innovation (Dubey et al., 2021; Mikalef et al., 2019; Wang et al., 2025) and market share (Mikalef et al., 2020). As an overarching concept, business analytics involves the use of data to carry out descriptive, predictive, and prescriptive analytics (Delen & Demirkan, 2013). Descriptive analytics focuses on using data to describe current and historical trends, whereas predictive analytics focuses on using historical data to make predictions (Delen & Demirkan, 2013). Prescriptive analytics focuses on the use of data to decide what action to take using techniques such as optimisation and simulation (Delen & Demirkan, 2013; Delen & Ram, 2018). Although substantial recent attention has been devoted to the more advanced forms of predictive and prescriptive analytics (Galetsi & Katsaliaki, 2020; Lepenioti et al., 2020; Smyth et al., 2024), descriptive analytics remains crucial in underpinning these higher levels of analytics maturity, thereby forming a foundation for advanced analytics. Business intelligence (BI) is central to descriptive analytics, providing interactive dashboards and reports to display key performance indicators numerically and visually (Delen & Ram, 2018). These solutions can now also incorporate more advanced forms of analytics, such as predictive analytics and generative artificial intelligence (Ruoff et al., 2023). Despite the importance of BI within the broader business analytics framework, it has received less attention in recent literature than advanced analytics.

Effective BI enables firms to gain a competitive advantage and make informed decisions (Mikalef et al., 2020). BI platforms focus on analysing and communicating data through reporting and dashboards. They allow firms to analyse current key performance indicators (KPIs) alongside historical trends, providing insights to improve decision-making and conveying these insights clearly and effectively (Li et al., 2022). Despite the recent development of advanced tools and techniques such as predictive and prescriptive analytics and generative artificial intelligence (AI), BI remains the most widely applied form of analytics and is particularly important in small and medium-sized enterprises (SMEs) (Ashrafi & Zareravasan, 2022) that may lack the more mature resources and capabilities needed to collect and analyse data effectively, or may not have the same strategic requirements as larger firms to utilise more advanced analytics tools.

A wide range of technical and organisational resources and capabilities have been identified as important in the development of business analytics solutions (Table S1, Supplementary Material 1) (Combita Niño et al., 2020; Corallo et al., 2023; Hausladen & Schosser, 2020; Lismont et al., 2017; Menukin et al., 2023). Specifically, data and their governance and management have been viewed as the core building blocks of analytics solutions (Corallo et al., 2023). Researchers also highlight the availability and quality of data as prerequisites for an analytics platform (Corallo et al., 2023; Hausladen & Schosser, 2020). Additionally, technical resources, including information technology and analytics infrastructure, are required to capture, store, manage and use data for knowledge creation and management (Comuzzi & Patel, 2016; Gökalp et al., 2021). The effective development and implementation of business analytics solutions also rely on organisational resources, including leadership, appropriate organisational structures, and employee skills (Cosic et al., 2015; Klievink et al., 2017). Although existing research has noted that relational resources are critical for successful development (Cosic et al., 2015; Menukin et al., 2023), there is limited empirical evidence on how to integrate CRM into the development of business analytics solutions (Phan & Vogel, 2010).

This study aims to integrate CRM in the development and implementation of a BI platform for a construction materials supplier in Northern Ireland (SDG^1^) as part of a university-industry knowledge transfer partnership (KTP) project^2^. KTPs are UK-wide programmes funded by Innovate UK^3^. They are public-private collaborative initiatives, involving a three-way partnership between a UK-registered company, a UK academic institution (the knowledge base), and a qualified graduate (KTP associate) (Gledson et al., 2024). SDG operates as a supplier in the construction industry, supplying building materials and other construction-related tools and supplies. As a business management KTP, the project aims to enhance SDG’s performance by addressing a specific business challenge and enabling the transfer of knowledge, technology, and expertise from the knowledge base to the company.

The project addressed the business problem of enhancing customer relationship management through business analytics and more effective strategic and operational planning. The overarching business aim was to improve customer engagement and retention, to drive sales and business growth. KTPs operate within a structured framework in which a KTP associate undertakes substantial work for a company under the guidance of industrial supervisors from the company and academic supervisors from the knowledge base. The company benefits from knowledge and expertise transfer, as well as access to new technologies, specialist facilities, and technical expertise (Ates et al., 2025). As practically orientated yet theory-informed projects with clear artefacts as deliverables, KTPs are an ideal setting for a design science study.

To guide the study, we propose the following research questions: How can CRM principles inform the design of BI platforms in SMEs? What design artefacts and design principles emerge from integrating CRM and BI in an SME context?

To address the research questions, we follow the design science research methodology, integrating CRM theory and practice with the design and delivery of the BI platform during the 24-month KTP project. Design science is a research paradigm that aims to design and develop practically useful, innovative artefacts, such as systems, models, and processes, as solutions to real-world problems and to create and validate prescriptive knowledge (Peffers et al., 2007; Tuunanen et al., 2024). With its focus on artefact development, design science research emphasises the generation of explanatory insights grounded in empirical evidence (Avdiji et al., 2020). This can lead to the development of new design knowledge, which may be useful across a broader context (Zolbanin & Aubert, 2025). The method has been applied to develop data analytics platforms and tools, such as visual inquiry tools (Avdiji et al., 2020), supply chain energy and carbon management systems (Zampou et al., 2022), and tablet applications for financial education (Zaitsev & Mankinen, 2022). The design problem for the study was to design and implement a solution to improve CRM through integrated strategic, process and technical business intelligence artefacts.

The study makes a practical contribution by demonstrating how to integrate CRM theory and practice with the development of a BI platform. The approach involves developing a data-driven customer strategy, embedding key account relationship development, and redesigning the customer engagement model, resulting in the formulation of strategic pillars across the organisation. Data-driven decision-making is facilitated by designing, developing, and implementing a BI platform that automates KPI analysis and dissemination through advanced visual analytics and automated reporting. As a result, the platform enables SDG to take a strategic, relational approach to understanding, managing, and engaging with customers and other key supply chain stakeholders. Implementing the platform has enabled SDG to move from reactive to proactive, data-driven decision-making, helping the organisation achieve its strategic objectives more effectively. The approach taken in this design integrates strategic, process, and technical artefacts, demonstrating that, in practice, both technical and organisational artefacts are necessary for CRM capability development.

We contribute to the literature by providing an innovative method and design principles for developing CRM-based BI (Gregor & Hevner, 2013). Most existing BI designs focus on technical artefacts, without considering the broader strategic and operational artefacts that should accompany them. Grounded in CRM, we propose a method that integrates the design of technical and organisational artefacts and the design principles that inform and guide the development of the BI platform. Hence, in this study, we improve on existing BI solutions by providing more efficient and effective decision-making support. The study demonstrates how BI can be used to implement and promote CRM in an SME, thereby improving understanding of the challenges SMEs face when implementing business analytics (vom Brocke et al., 2020). The findings also inform the development of a CRM-guided design process. This study contextualises BI development within an SME in the construction industry, enhancing the fit of the design knowledge (vom Brocke et al., 2020).

## Literature review

The study is grounded in the literature on BI platform design and CRM. We begin by reviewing related work on BI platform design, followed by the relevant CRM literature. This is followed by the development of an integrated CRM-BI framework, which guides the design of the overall solution.

### Business intelligence

BI platforms encompass the processes, tools, and technologies used to transform data into information, and information into knowledge and actionable plans that drive effective business activities (Eckerson, 2012). BI delivers relevant information to the right people at the right time, enabling managers to act quickly and efficiently. BI platforms typically include ‘back end’ components to store and process data from underlying source systems, such as data warehouses, as well as ‘front end’ analytics, dashboard and reporting tools which allow end users to view and interact with the analytics (Duque et al., 2022). Traditionally, information derived from data has been disseminated across companies through standard reporting, which presents a static view of KPIs. However, more recent approaches centre on interactive self-service dashboards, which allow an end user to view current and historical data and to interrogate it using various filters and selectors. The communication of data insights can be efficiently managed through these dashboards—defined as visual displays that consolidate and organise the most important information required to achieve one or more objectives on a single screen—thereby enabling effective monitoring (Recker, 2021; Ruoff et al., 2023). Using charts, graphs, and numerical summaries, a BI platform can transform large, complex datasets into meaningful visual representations. An effective dashboard can also accelerate organisational decision-making and sensemaking for respective audiences, monitor performance outcomes, and enhance overall productivity (Eckerson, 2012).

Studies have applied design science to the development of BI platforms across various fields. For example, Kao et al. (2016) developed a BI platform for hospital decision-making, identifying benefits for the organisation and highlighting the importance of top management support. Recker (2021) analysed the in-situ implementation of Germany’s RKI COVID-19 Dashboard to develop new design principles to extend the state-tracking capabilities of COVID-19 dashboards. Ruoff et al. (2023) designed a crisis response dashboard by integrating natural language interaction capabilities to improve transparent user interaction and access to crisis-related information.Vо and Plachkinovа (2023) built a data-driven dashboard that helps judges make fairer, more objective decisions by integrating diverse data points. Amirasari and Andrias (2024) developed a BI dashboard artefact for analysing Employee Engagement Survey data within an organisation. Gledson et al. (2024) developed a digital, web-based dashboard prototype to enhance the efficiency, productivity, and effectiveness of construction design management. These studies confirm the usefulness of the interactive components in the BI platform and provide empirical evidence that they generally enhance user engagement with the dashboard and make it more accessible to a broader audience. Organisations may face challenges in creating effective, interactive, data-driven dashboards due to inadequate stakeholder engagement and relationship-building during the design stage. However, there is limited guidance on integrating CRM using an interactive, relational approach in platform design and development.

### Customer relationship management

CRM focuses on the processes and systems for managing customer interactions, with the aim of increasing customer retention and profitability by enhancing loyalty and satisfaction (Aziah et al., 2009; Khodakarami & Chan, 2014; Nguyen et al., 2007). CRM approaches enable firms to interact effectively with customers and to collect, store, and analyse customer data, thereby generating useful knowledge about customers, which can be used to enhance relationships (Khodakarami & Chan, 2014). CRM systems and processes store and manage information on customer interactions, including customer details, quotes, sales, complaints, tastes and preferences, and other customer-related communications (Nguyen et al., 2007).

Alongside these systems and processes, it is important to ensure that CRM aligns with the wider business strategy, considering factors such as current and potential customers, the desired relationships with these customers, opportunities for growth, the firm’s internal and external environments, and technology requirements (Frow & Payne, 2009). Businesses can also implement CRM, considering factors such as the degree of individualisation and the information they need to collect about customer requirements, leading to a range of specific CRM approaches, including product-based selling, managed services, customer-focused marketing, and individualised relationship management (Frow & Payne, 2009). CRM can help businesses to better understand customers, build relationships, and target profitable customers, thus increasing customer satisfaction and loyalty (Lee & Park, 2005; Nguyen et al., 2007). Effective CRM is important to retain and drive growth from existing customer relationships, as it is often more expensive to acquire new customers than to maintain existing customers (Phan & Vogel, 2010).

### Integrated CMR-BI framework

CRM has been conceptualised as focusing on multiple business areas and functions. For example, Zablah et al. (2004) posit that CRM has been conceptualised as focusing on technologies, business processes, and/or strategies. Similarly, Chen and Popovich (2003) posit that CRM focuses on people, process, and technology. It has also been recognised that CRM involves all business functions, including sales and marketing, as well as other functions such as operations, finance, R&D, and human resources (Chen & Popovich, 2003). These conceptualisations of CRM highlight strategic, process, and technological aspects of CRM across business functions.

Strategic CRM focuses on the allocation of resources to develop customer relationships to maximise customer lifetime value management (Zablah et al., 2004). CRM processes place more emphasis on the changing relationship with customers, and focus on the business processes that convert CRM inputs to outputs (Chen & Popovich, 2003; Zablah et al., 2004). CRM processes include knowledge management and interaction management (Zablah et al., 2004). The knowledge management process takes data as input, transforms it into insights, and disseminates them, which then feed into interaction management processes (Zablah et al., 2004). Interaction management processes can include customer evaluation, for example, using customer lifetime value, as well as interactions with customers (Zablah et al., 2004).

Technology has played a prominent role in CRM, and includes the range of technologies needed to acquire, process, store, and analyse data relating to customer relationships. Despite most design science studies focusing primarily on the technical artefact, the importance of an integrated approach that incorporates strategy, organisational processes, and technology has been recognised in the wider literature (Chen & Popovich, 2003; Zablah et al., 2004). Focusing solely on technology when implementing CRM is unlikely to improve business performance (Chen & Popovich, 2003). Rather, CRM technologies need to be combined with other organisational capabilities and processes to enhance performance (Chang et al., 2010).

**Fig. 1:**
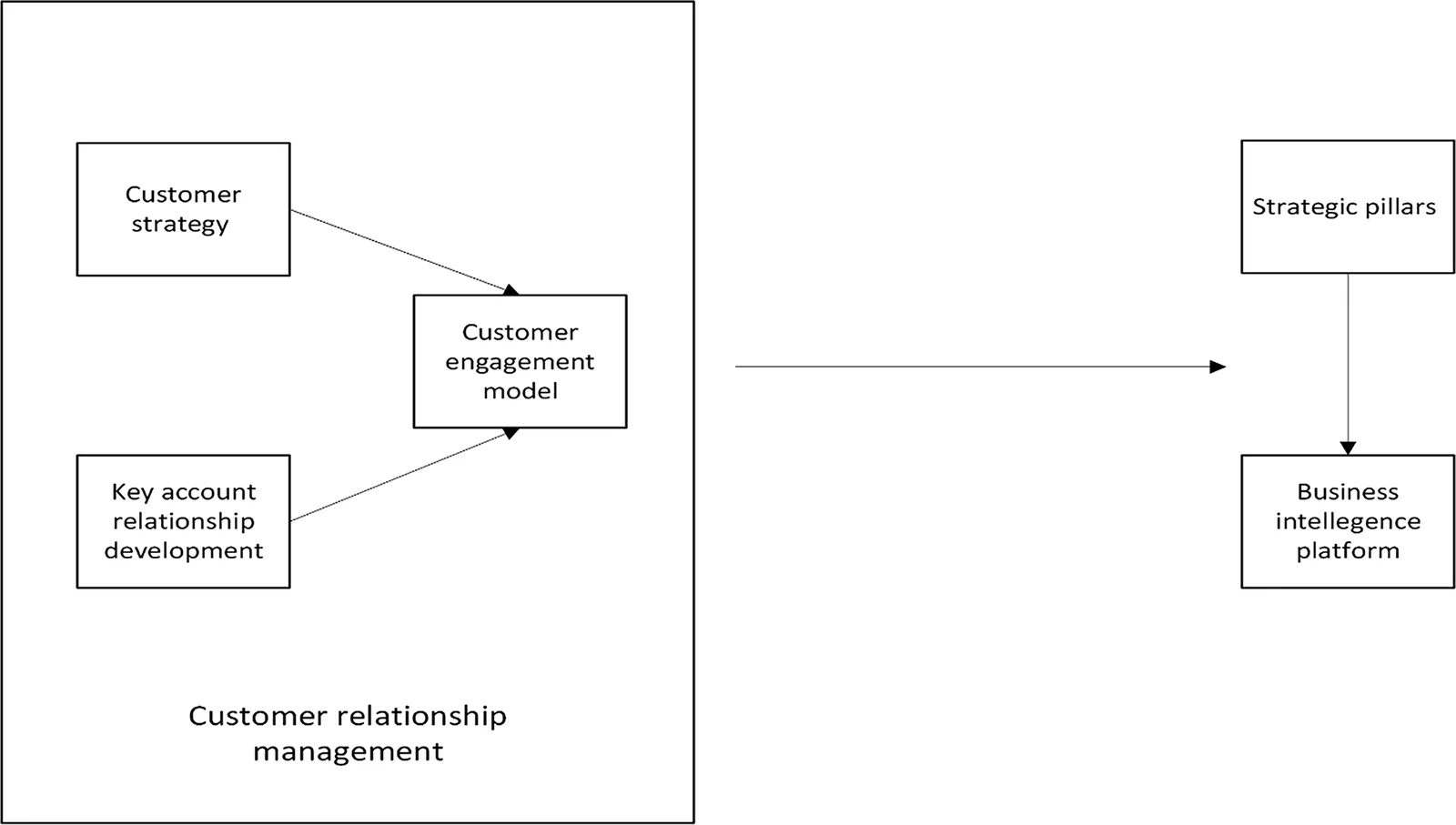
The CRM approach for BI platform development

We draw on these conceptualisations and the wider CRM and BI literature to develop an integrated CRM framework which includes strategic, process, and technological components. This framework, presented in Fig. 1, guides the design of our overall CRM-BI solution. As shown in Fig. 1, the framework integrates SDG’s overarching strategic pillars, CRM strategy and processes, and the BI platform. The framework demonstrates how CRM guides the design, development, and use of the BI platform, leading to more effective data-driven decision-making. Additionally, SDG’s strategic pillars are informed by CRM and, in turn, shape the development of the BI platform. The following paragraphs explain how these elements are interrelated.

Our framework includes strategic CRM artefacts, centring on the customer strategy and the organisation’s strategic pillars. Alongside these strategic artefacts, the framework also includes the technical BI platform and process artefacts, including key account relationship development and the customer engagement model. Key account relationship development focuses on analysing and mapping current customer relationships and setting targets for each account. This process helps to identify opportunities and risks. The key account relationship development and customer strategy feed into the customer engagement model. The customer engagement model focuses on the collection and analysis of data across the customer journey – identifying opportunities for upselling, customer satisfaction, and improving relationships. In line with the literature indicating that implementing CRM requires cross-functional change (Chen & Popovich, 2003), a service blueprint is developed to outline CRM activities across business functions.

The framework also incorporates strategic CRM elements, focusing on the overall customer strategy, alongside the wider business strategy. The customer strategy includes customer segmentation and prioritisation to enable the company to focus on customers with high growth potential. CRM is also central to the overall business strategy, which we formulate as ‘strategic pillars’ focusing on growth, innovation, customer centricity, operational excellence, and working environment.

These CRM processes and strategy and overall business strategy then inform the design of the BI platform, which falls within the technology aspect of CRM conceptualisations, as well as supporting the knowledge management CRM processes (Chen & Popovich, 2003; Zablah et al., 2004). The BI platform includes data acquisition, processing and storage, as well as the front-end BI dashboard and reporting to disseminate insights to business users. The main data storage aspect of the BI platform is the data warehouse. The literature highlights that a wide range of CRM-relevant data can be integrated into data warehouses, including order information, returns, customer interactions, customer satisfaction, and data related to the customer’s business (Chen & Popovich, 2003). This data can be obtained from a variety of sources, including internal systems, surveys and feedback, social media and other web sources. When integrated and presented effectively in BI dashboards, this can improve understanding of customers across the customer journey, resulting in improved decision-making and better relationship management.

The importance of BI in CRM has been recognised in the literature (Chen & Popovich, 2003). BI can complement CRM systems and processes by providing the analysis and information needed to enhance customer satisfaction and drive business success (Phan & Vogel, 2010). The integration of BI and CRM can help segment and profile customers, identify profitable customers, and facilitate the measurement and monitoring of CRM performance (Habul & Pilav-Velic, 2010). Although the importance of BI in CRM has been recognised, less is known about how CRM approaches can be combined with the development of BI platforms to support firms’ growth. Whilst the CRM and BI platform design literatures provide the theoretical underpinnings for the study, they do not provide guidance on how to combine these two areas to design and develop useful artefacts.

Ultimately, the aim of CRM is to build relationships with customers, with the technology supporting this aim by enhancing the understanding of customers (Chen & Popovich, 2003). To meet this aim, BI dashboards must provide the information needed to improve customer understanding and relationship building. This means the dashboard design should be driven by CRM needs. This is likely to require close interaction between the design of strategic and operational CRM artefacts and technical CRM artefacts, rather than designing either in isolation.

CRM integrates data across customer touch points to gain a better understanding of customers (Chen & Popovich, 2003). More generally, a range of business analytics applications can be applied to data collected across the stages of the customer journey to support CRM. This can include applications such as segmentation, customer profiling, churn prediction, dissatisfaction analysis, and product recommendation (Zerbino et al., 2018). These applications rely on data and can be applied across stages of the customer journey from relationship initiation, through to maintenance and termination (Zerbino et al., 2018). This highlights the need for a close connection between information collected across the customer journey and the technical BI platform. Sharing CRM data across the organisation can also yield significant benefits, such as identifying opportunities for upselling, improving customer service, enhancing understanding of customers, and enabling segmentation and targeting (Chen & Popovich, 2003). A CRM-informed BI solution can help disseminate this information to realise these benefits.

To summarise, our CRM-BI framework highlights the need to integrate strategic, process, and technological artefacts in the development of CRM-BI solutions. The CRM strategy and processes inform the data, information, and insights that are needed to effectively implement CRM. CRM is a core aspect of the organisation’s strategy and therefore also informs the information required for the BI platform. The technical BI platform presents this information to end users through dashboards, supporting more effective decision-making and ultimately enhancing customer understanding and relationship management. Despite this, past design science studies on BI have focused on technical artefacts, without considering the wider strategic and process factors that shape BI design. We therefore take a more integrated approach than past literature when designing our CRM-BI solution.

## Method

We draw on the design science research methodology (Peffers et al., 2007) to study how CRM can be combined with the development of a BI platform to improve firm performance. In contrast to studies focusing primarily on theory development or testing, the design science methodology focuses on gaining useful knowledge through the design of practical artefacts intended to solve organisational problems (Hevner et al., 2004; Holmström et al., 2009). Rather than developing or testing new theories, design science artefacts draw on kernel theories, which are bodies of existing knowledge, to explain why an artefact should work (Hevner et al., 2004; Iivari, 2020). Although the literature includes a range of approaches regarding design science methodology, commonly applied stages in the process include problem identification, setting objectives, solution design and development, demonstration, evaluation, and communication (Peffers et al., 2007).

In implementing the design science method, we first identified the issues faced by SDG and the aims and objectives of the KTP project. The project aims and objectives were identified and refined through initial discussions between SDG and the academic team. A formal project work plan was developed from these initial discussions, which detailed the design requirements and activities to be carried out over the two-year project. The activities in the workplan were agreed by the business and academic partners, with input from the KTP advisor and the University KTP office. This workplan was reviewed by the funder, who subsequently funded the project. The ethics of the project were also considered at this stage in line with University ethics and integrity standards and guidelines. Data analyses were carried out within the business by the KTP associate, maintaining confidentiality and privacy. Once funding was received, a recruitment process was conducted to hire the KTP associate. The KTP associate is based at the company and undertook most of the artefact design and development, with support from the academic team and company personnel.

As presented in our overarching framework and included in the project workplan, the project involved the design and instantiation of strategic, process, and technological artefacts. These include the customer strategy, key account relationship development, customer engagement model, overarching strategic pillars, and the BI platform. Together, these artefacts make up the overarching CRM-BI solution. The artefacts were designed in collaboration among the business, the academic team, and the associate, with the associate primarily responsible for their practical development and implementation. Evaluation of design science artefacts is important to gain feedback, as well as to provide rigour to the research process (Venable et al., 2016). Evaluation of the artefacts included both formal and informal evaluation, and was both formative and summative (Venable et al., 2016). This use of multiple sources of formative and summative evaluation evidence at different stages of the project is another important aspect of design science research evaluation (Venable et al., 2016), which we have incorporated in our evaluation approach.

The artefacts were formally evaluated on an ongoing basis during the project through a series of six local management committee (LMC) meetings, each lasting for around one hour. Each meeting included the following people: the KTP associate, the company supervisor, the Chief Executive of SDG, the two academics involved in the project, a KTP advisor from the funder, and one administrative staff member. This group provided expert feedback on each artefact from differing perspectives. Prior to each LMC meeting, the associate, academic team, and company team were required to submit a written report detailing progress to date. During the meeting, these reports were presented to the committee. The focus of each meeting was the associate’s presentation, which detailed progress to date against the original workplan and presented or demonstrated any artefacts produced since the previous meeting. This included presenting the strategic and process artefacts and demonstrating the technical BI solution. The meeting committee provided feedback on each artefact, leading to ongoing refinement throughout the project. Detailed minutes were also taken during the meeting.

As the associate was based in the company, more informal evaluation of the artefacts occurred on an ongoing basis from company personnel and management. Following completion of the project, an overall formal evaluation took place. This consisted of a comprehensive final evaluation report, with sections completed by the academic team, the company, and the KTP associate. The final reports contained a series of 50 questions that required both qualitative and quantitative evidence of the project’s performance. The associate’s sections contained questions on the original project objectives; changes to the original objectives; the main activities, milestones, and outcomes; changes to the project plan and lessons learned; project effectiveness and satisfaction with the project; assessment of others’ satisfaction with the project; additional post-project resources required to maintain the benefits; summary of documents logged with the company at project closure; details of training provided to the company; whether the project met expectations; and suggested improvements to the KTP. The partners’ sections included a jointly completed summary of the overall project, followed by questions to be completed separately by the academic and industry partners. Questions for the company were both quantitative and qualitative, assessing the project’s outcomes and its impact on company performance. Questions related to: business changes arising from the project; contribution of the project to the company strategy; external factors influencing company strategy or the KTP project during the project; the business opportunity addressed by the project; new knowledge and skills acquired by the company’s staff from the project; how these skills and knowledge were embedded in the company; immediate and forecast changes in sales, exports, and profit resulting from the project; other enhancements to the company’s operations from the project, social and cultural benefits; the nature and significance of the impacts; investments to exploit the results of the project; future impacts of the project on company performance; and whether the project matched up to expectations. Questions for the academic team were also a mix of qualitative and quantitative, with a greater focus on the project’s academic benefits. Questions for the academic team focused on academic staff development, impact on teaching, postgraduate projects from the project, research benefits, other institutional benefits, and whether the project exceeded expectations. In total, the completed report contained 23 pages of evidence that was used to inform the formative ex-ante evaluation of the project artefacts.

The questions in these evaluation reports are designed by the project funder and have been used extensively to evaluate other KTP projects, indicating both their practical utility and their reliability as evidence for evaluation. Following submission of the final project report, the project was formally assessed by independent KTP advisors who were not involved in carrying out the project. Although the exact criteria for this final evaluation are not made available, the project received the highest rating of ‘outstanding’, further indicating the project’s overall success. The use of formative and summative evaluations from multiple sources during the design and evaluation of the project helps triangulate evaluation evidence, thereby increasing rigour in the design science process.

## Artefact description

The project involved designing five artefacts, with the key results focusing on the artefact design and description, and design principles. Strategic and process artefacts include the customer strategy, key account relationship management, customer engagement model, and the organisation’s overall strategic pillars. The focal technical artefact is the CRM-BI platform, central to which is the CRM-BI dashboard. We first present the company background and project objectives, followed by the description and design of each artefact.

### Company and project background

SDG is a family-owned SME and one of the main suppliers of specialist construction materials to the building and construction industry in Northern Ireland and the Republic of Ireland. SDG’s mix of product quality, rapid delivery, dependability, competitive pricing, and commitment to customer service has enabled it to build a client base ranging from SMEs to blue-chip construction companies, public bodies, and government departments. Its vision is to become a £50 million business, bringing innovation and high-quality jobs to the local economy. The KTP team comprises the KTP associate, who holds a master’s degree in business analytics, two industrial supervisors (i.e., SDG’s Chief Executive and Finance Manager), and two academic supervisors. The KTP associate was based in SDG for the duration of the twenty-four-month project, and the two academic supervisors spent an average of half a day per week on the project. The academic team's involvement included meetings with the company management, the associate, and the funder, as well as input into the solution’s development. Close interaction with the company over this period provided the academic team with detailed insights into the project and the organisation, thereby facilitating the design science method used in this study.

### Problem identification and project objectives

Before the project began, SDG had already established routes to market through construction companies, builders’ merchants, and distributors. SDG conducted a strategic review that identified a need to shift from product marketing to relational marketing. The review highlighted the need to understand customer needs and consistently deliver on them, alongside ongoing KPI monitoring and measurement of CRM initiative impact. Additionally, SDG had a limited understanding of how to partner more effectively with customers and identify their key issues and needs. SDG recognised that customer engagement was ad hoc and intermittent, rather than organised through an effective CRM process. This created an opportunity to improve customer engagement, enabling SDG to meet customer needs better and drive sales. Hence, analysing gaps in relationship marketing and identifying where SDG might develop opportunities was critical to achieving its growth objectives. The review also highlighted a significant skills gap in data-driven decision-making and a lack of internal expertise to develop and implement advanced analytics solutions. Operational inefficiencies were another pressing issue, as manual processes consumed valuable time and resources, thereby limiting productivity and overall performance. Moreover, the existing business structure created bottlenecks, preventing senior staff from dedicating time to strategic initiatives. SDG lacked the knowledge and tools to enable the senior management team to make data-driven decisions.

The KTP project was motivated by the need to improve customer relationships and by the recognition that data and analytics could enhance the business. Given these challenges and opportunities, the overarching aim of the KTP project was to enhance business growth by leveraging the latest thinking in CRM and business analytics. This was paired with a more strategic, proactive approach to understanding, managing, and engaging with customers, enabling SDG to shift from reactive measures to a more proactive, data-driven strategy. Although SDG had a good understanding of the underlying issues, it lacked a clear vision for addressing them. The company also understood that business analytics could help drive business objectives, but lacked in-house expertise to use it effectively and instead relied on basic spreadsheet analyses and intuition-based decision-making. The academic team, therefore, worked with SDG to develop a project plan to integrate CRM with a data-driven BI solution. Specifically, the project designed a CRM-based BI platform that enables SDG to access real-time, on-demand data and analytics, a comprehensive data governance framework, and strategic and process artefacts. Development of the CRM-BI platform involved developing mechanisms for data delivery and presentation, including designing the data flow and storage, modelling the data, implementing security, monitoring and modification, and creating a new interactive reporting and visualisation interface.

### Artefact design

The focal artefact for the study is the CRM-BI platform. As presented in our framework, the design of BI platforms occurs within a broader strategic and organisational context. The CRM-BI platform is therefore informed by the development and instantiation of strategic and process artefacts, including the customer strategy, key account relationship development, customer engagement model, and strategic pillars.

#### Customer strategy

The first part of the CRM approach involved developing a customer strategy by investigating current customer interaction and purchase behaviour. We created a customer strategy for existing customers by conducting a detailed analysis alongside cluster analysis for customer segmentation. This enabled us to identify customers with significant growth opportunities based on current engagement levels and customer financials, with the overall aim of increasing SDG’s growth. We then selected a sample of customers to conduct a detailed analysis of their relationships, interaction types, and frequencies. We also examined customer purchasing behaviour to identify reasons for sales declines, which informed improvements in account management and communication. Customer lifetime value was also calculated using a historical approach based on sales revenue from 2017 to 2020 to identify where relationships were weakening and could be strengthened. A product purchase trend analysis helped investigate reasons behind significant sales drops for certain products. An example of customer analytics is provided in Fig. S1, Supplementary Material 1, offering insights into KPIs for customer relationship management. The findings of this analysis were presented in a workshop to gather employees’ views on the criteria for customer segmentation and prioritisation and on monitoring and measuring customer lifetime value.

**Fig. 2:**
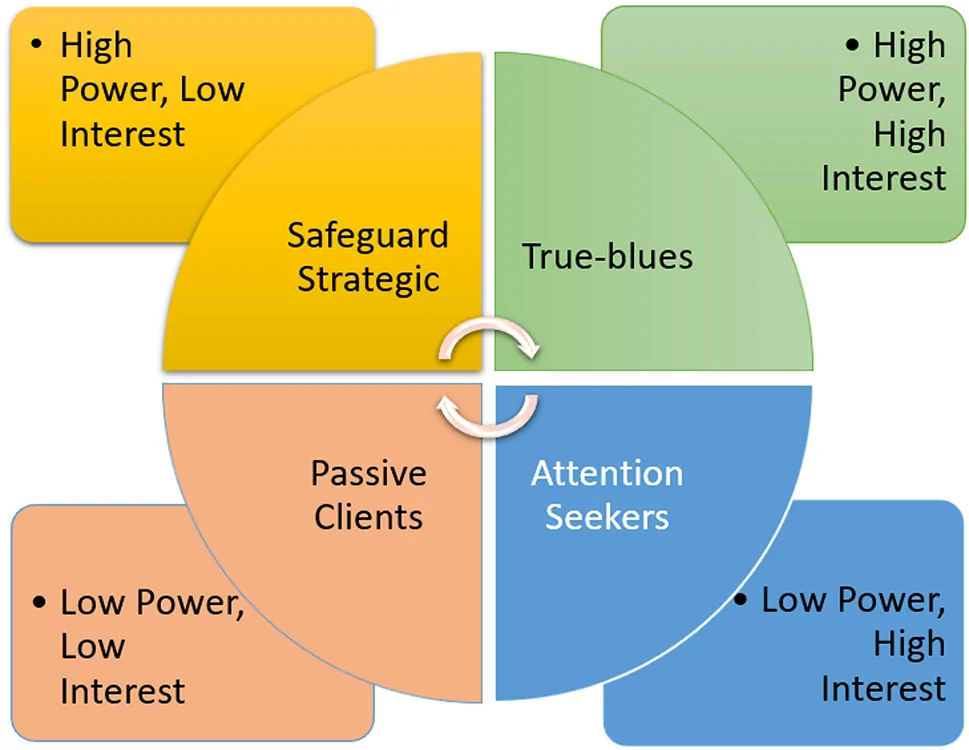
Customer segmentation

Customer segmentation was undertaken using k-means clustering. Data for the cluster analysis are from 302 customers and include variables such as customer lifetime value, order frequency, company turnover, sales in the past 12 months, total years in business, and total years with SDG. This resulted in four customer segments as outlined in Fig. 2, with descriptive statistics for each cluster presented in Table S2, Supplementary Material 1.

Detailed strategies were developed to manage relationships with each customer segment. Feedback from the business indicated that it would be useful to further subdivide customers based on relationship strength into supporters, neutrals, and defectors. Supporters are loyal customers who maintain a supportive, nurturing relationship with SDG and prefer to do business with SDG over competitors. Neutral customers tend to be indifferent to whether they do business with SDG or its competitors, and although they do work with SDG, they are more likely to ‘shop around’. Defectors are customers at high risk of churning due to their weaker relationship with SDG. Specific strategies were then developed for each customer segment, broken down further by relationship strength. A detailed overview of the specific strategies developed during the project and used by the business is presented in Table S3, Supplementary Material 1.

The first segment includes ‘True-blues’. These are larger customers who generate long-term repeat business and account for around half of SDG’s total revenue. They are highly engaged customers with significant power and influence and should therefore be managed carefully. SDG relies heavily on these customers and maintains strong relationships that it aims to sustain and enhance. To support these relationships, SDG plans to communicate regularly with these customers. For supporters, this can include informal contacts to build relationships, as well as providing training and information on product updates. For neutrals, actions can focus on formal updates, occasional informal communications, and training. For defectors, efforts focus on ensuring concerns are recognised, gaining a better understanding of satisfaction levels and current projects, and providing technical advice.

The second segment comprises ‘safeguard strategic’ customers. These are customers who do not currently generate as much revenue as true-blue customers but have the potential to increase their revenue through stronger relationship-building. These customers hold significant influence, and careful relationship management should be employed to ensure they remain with the business. For example, additional relationship building may be required to boost sales and prevent churn. For supporters, CRM actions can focus on providing product updates and building relationships. For neutrals, actions can again focus on keeping customers informed and addressing any concerns. For defectors, actions can focus on addressing concerns and on targeted efforts to win customers back.

The third segment includes ‘attention seekers’. This group comprises a diverse range of smaller businesses with limited influence, but together they generate a significant portion of SDG’s revenue. These customers require a high level of relationship management through regular communication to sustain and grow sales. This involves addressing any product queries they may have. To maintain an effective relationship with this customer group, SDG aims to ensure consistent contact. While communication is important, it is carefully managed to balance efficiency, relationship-building, and sales growth. For supporters, actions can include maximising opportunities for bulk orders to increase efficiency, as well as effective account and time management. For neutrals, actions focus on improving customer satisfaction, providing product-related education, and managing relationships by geographical area. For defectors, actions can focus on addressing concerns, ensuring appropriate pricing, and educating about SDG service and values.

The fourth segment includes ‘passive clients’. These are customers with low power and low interest. To maintain an efficient and effective relationship with this customer group, SDG aims to monitor their needs and avoid over-investing time. Marketing efforts for these clients are more focused and targeted. For supporters, this can include general marketing communication and responsive account management, as well as specific marketing communications as necessary. For neutrals, this can also include general communications and individual-level interaction as required. For defectors, actions can focus on allaying concerns through general channels and responsive sales contact.

#### Key account relationship development

As shown in Fig. 1, the second element of the CRM approach involved developing key account relationships. We created two templates to collect quantitative (customer data) and qualitative (relationship map) information for analysing the development of key account relationships (Fig. S2, Supplementary Material 1). First, we examined the relationships within the account, mapping all customers and relevant stakeholders. This information helps SDG identify which relationships to build and maintain, as well as identify customers who could potentially disrupt plans. Detailed data were gathered, including individual titles, decision-making roles, the extent of current communication, and the level of friendliness. Second, after collecting the data, we analysed the customer’s current business processes, objectives, and financial health. This analysis adds value to the account, helps identify mutually beneficial opportunities, and allows SDG to gain a comprehensive understanding of the client’s business, including current goals and initiatives. Third, we set targets for the account, including current account value, opportunities won and lost, potential revenue growth, revenue forecasts, and short-, medium-, and long-term goals.

We then collaborated with the senior management team to develop a strategy for strengthening key account relationships. The strategy aims to build long-term, mutually beneficial partnerships with important customers. It helps account managers identify opportunities to create value for their partners by diagnosing issues, proposing innovative solutions, and leveraging partnerships to guide the organisation toward its strategic objectives. It seeks to foster customer loyalty, encourage growth, and boost profitability, while driving innovative, scalable service solutions, shortening sales cycles, reducing acquisition costs, and prioritising relationships.

#### Customer engagement model

As shown in Fig. 1, the customer strategy and key account relationship development feed into a customer engagement model, which was developed by analysing the current customer experience and gathering data at each stage of the customer journey, including customer interactions and service levels. Specifically, we collected data from external sales (order completion and fulfilment), warehousing and delivery (stock, products, and deliveries), and internal sales (follow-up). To refine the customer journey and KPIs, we conducted a workshop and individual meetings with employees across departments (marketing, sales, finance, and warehousing and delivery), and observed employee-customer interactions (with delivery drivers and the sales team).

We segmented the customer journey into five stages (Awareness, Consideration, Decision/Service, Engagement, and Loyalty) and outlined the main goals, the data that can be collected, and the insights to be gained at each stage (Table 1). We also created a framework to gather customer insights throughout the journey (Fig. S3 in Supplementary Material 1). This framework details the points at which insights should be gathered across the purchasing process, specifying the necessary level and type of analysis. The customer insights are intended to help SDG boost cross-selling and upselling and identify opportunities to improve customer satisfaction, ultimately fostering greater customer loyalty.

**Table 1:** The customer journey

Stage	Goal	Data Sources	Insights gained
Awareness	Contact acquired	Customer contact and business information from websites, brochures, social media, and direct inquiries.	Number of business development opportunities.
Consideration	Product signup	Telephone enquiries, email inquiries, Construction Industry Scheme tender notifications and quotes.	Potential leads information, interested products list.
Decision/Service	Value delivered	Point-of-sale transaction information (customer, products, price). Order confirmation form. Invoice information. Dispatch report. Proof of delivery document.	Product sales trends. Current and historical purchases by customer. Order fulfilment times.
Engagement	Fully engaged	Follow-up logs, Satisfaction and engagement scores, Sales information.	Level of engagement indicated by customer feedback, customer satisfaction and engagement, repeat transactions.
Loyalty	Grow the lifetime value	Referrals, Testimonials and feedback, Net promoter score.	Customer loyalty is indicated by business value arising from referrals, positive testimonials and feedback, and a high net promoter score.

**Fig. 3:**
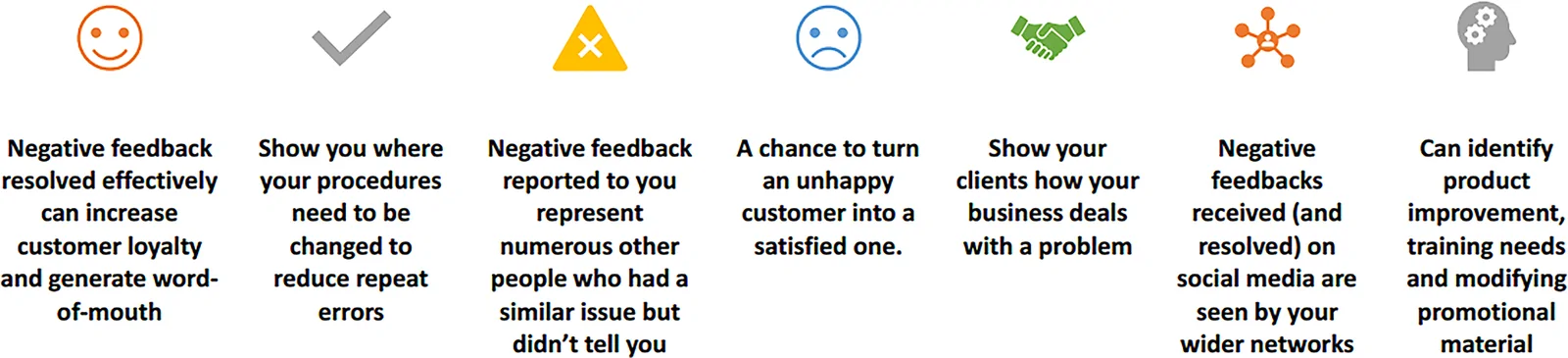
The classification of negative feedback

We developed two methods to collect customer feedback. The first is the customer satisfaction survey (Fig. S4, Supplementary Material 1). Surveys are used to identify customers’ pain points and assess their satisfaction with SDG’s products and services. Specifically, the first half of the questionnaire focuses on customers’ perceptions of SDG’s current offerings, while the second half explores areas for improvement. We also included the Net Promoter Score to measure overall customer satisfaction and loyalty. The second method involves ‘feedback and feed-forward’ (Fig. S5, Supplementary Material 1), which involves customer feedback received via phone, email, and meetings. Negative feedback is acknowledged immediately and assigned to the responsible individual for resolution within 48 h. A system to classify negative feedback has been developed (see Fig. 3), ensuring that SDG both acknowledges and promptly addresses any issues. We also established methods to act swiftly on customer feedback through automated processes and personal interactions. Specific metrics are collected during feedback, including contact details/role, date of feedback, channel used, reason for dissatisfaction, product details, responsible person, actions taken, status, and date of resolution. The goal of collecting feedback is to understand customer needs, improve and develop better products, and foster customer loyalty by serving their needs more effectively. Feedback also enables SDG to innovate in response to customer needs and to strengthen its overall brand and value proposition.

**Fig. 4:**
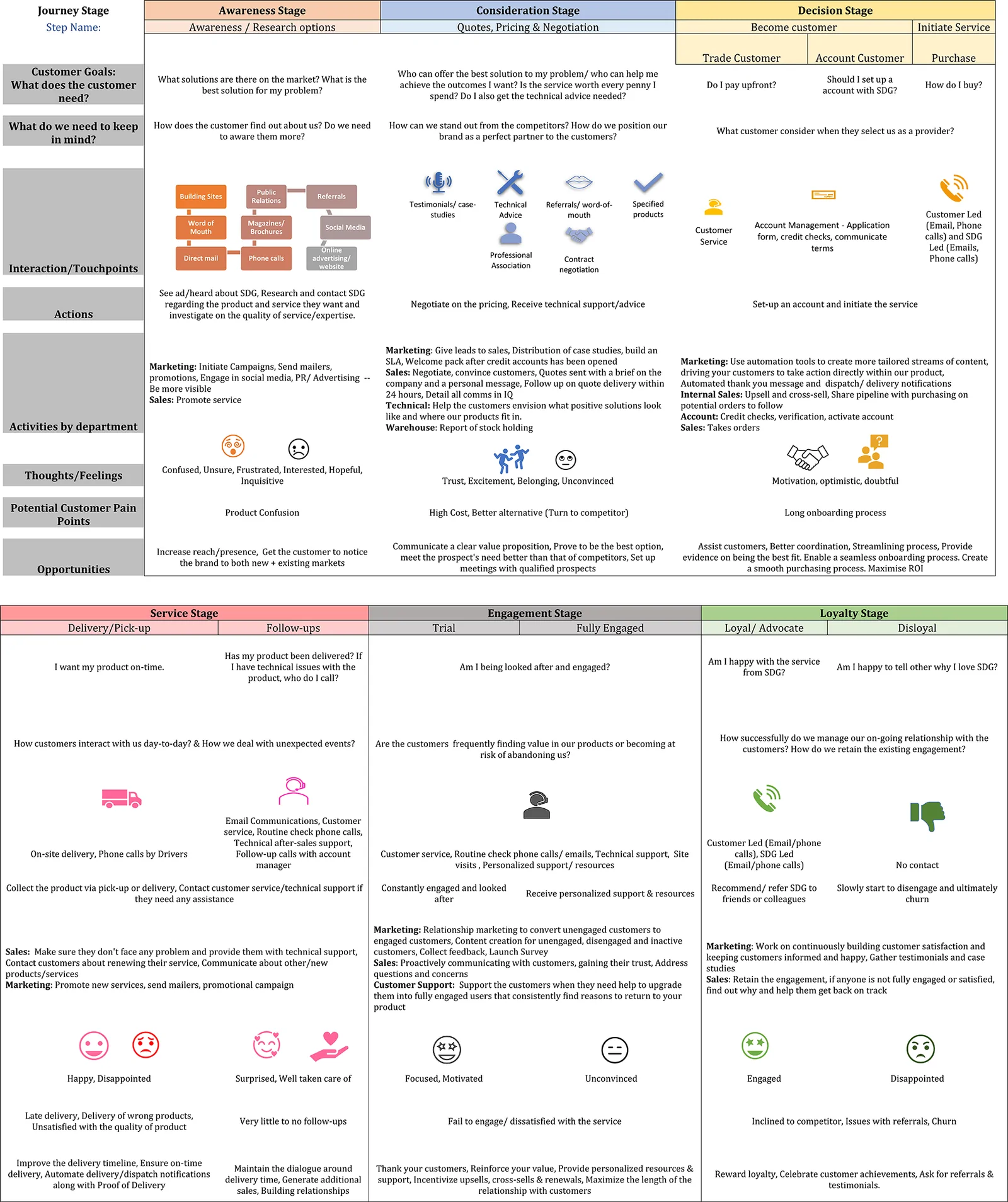
Customer engagement model

We created a service blueprint to outline the activities of SDG’s departments (i.e., Marketing, Internal Sales, Sales, Technical Department, Accounts, and Warehouse/Delivery/Purchasing teams), covering both customer-facing and ‘back office’ operations, as well as support processes throughout the customer journey. We then developed a customer engagement model for SDG based on the customer journey and service blueprint (Fig. 4). This engagement model summarises and highlights key findings from the CRM approaches undertaken during the project, including customer needs, major action points, customer interactions and touchpoints, customer actions, departmental activities, customer thoughts and feelings, potential pain points, and opportunities for improvement at each stage of the journey.

#### Strategic pillars

**Fig. 5:**
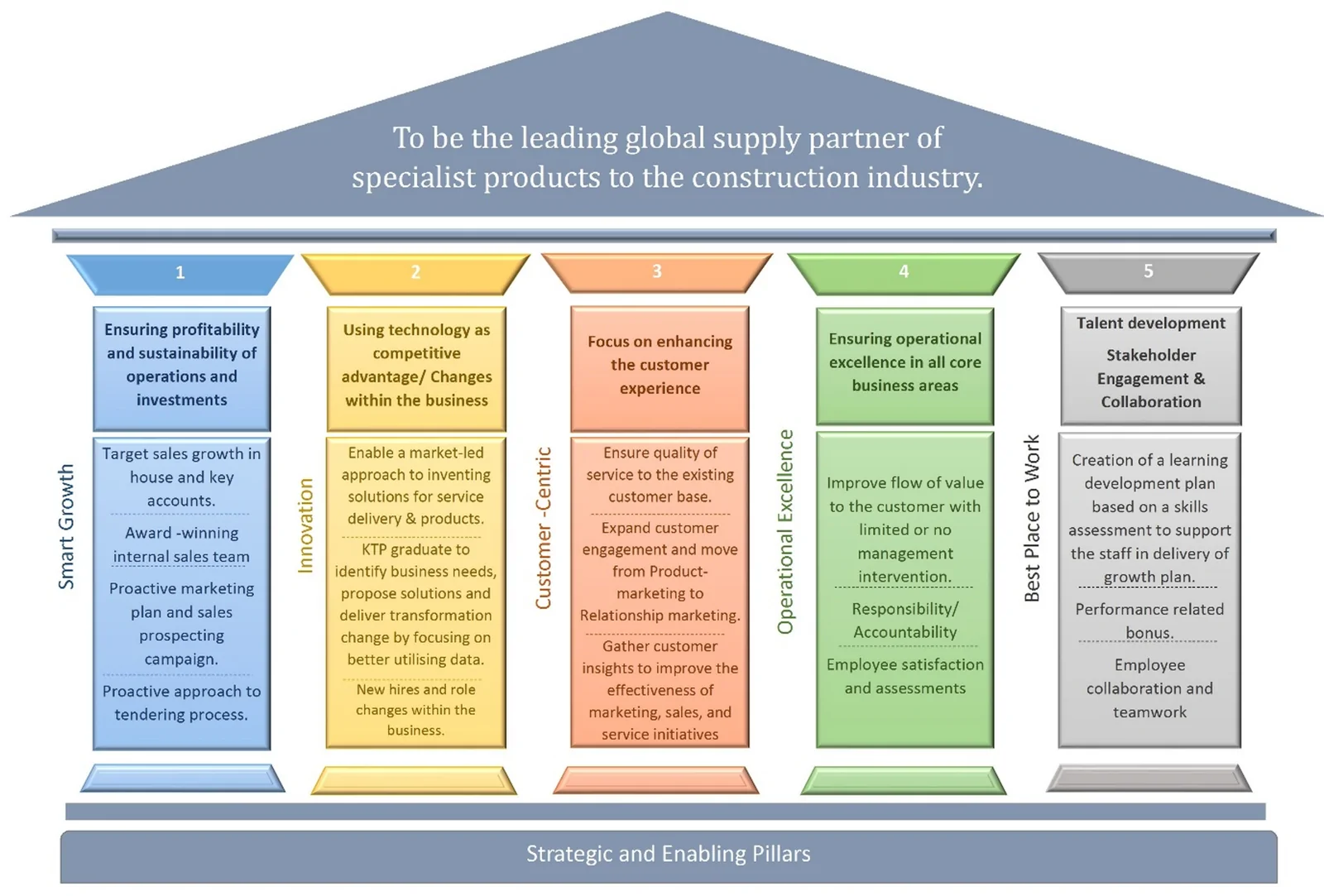
Strategic pillars

As shown in Fig. 1, the CRM artefacts inform the strategic pillars and the design of the BI platform. These elements are closely interconnected in creating the overall solution. To define the strategic pillars, the academic team collaborated with SDG’s senior management team to develop a company strategy structure (i.e., Strategic pillars) that supports the implementation of the 5-year strategic plan (Fig. 5). Among the five pillars, Pillars 1, 2, and 3 are particularly relevant in highlighting the need for a BI platform, with Pillars 1 and 3 both pointing to the requirement for CRM business analytics, and Pillar 2 explicitly highlighting the better use of data as a core element of the SDG’s innovation strategy. For example, the strategic objective of smart growth in pillar 1 can be supported by segmenting target customers into clusters and leveraging data to understand customer buying behaviour and product purchase patterns throughout their customer journey. Focusing on customers with significant growth potential will help SDG reach its overall growth goals. A customer-centric approach prioritises improving customer experiences. SDG can ensure quality of service and enhance the customer experience by understanding and delivering on customer motivations and continuously tracking impact through engagement.

#### Business intelligence platform

A workshop and meetings with the company’s senior management team were organised to gather information to inform the design of the BI platform. The KPIs to be tracked were jointly decided with the senior management team and used to shape the report and dashboard layout. We reviewed SDG’s existing data architecture to identify issues and risks and to implement role-based security for reports. The BI platform aims to deliver and present insights effectively. From a technical perspective, this involved designing the data warehouse, modelling the data, implementing security measures, monitoring and adjusting data, and creating a new dashboarding and reporting tool. This directly supported SDG’s overall business strategy. During development, we extracted the necessary data from source systems, stored it in a SQL Server data warehouse, and used Power BI to build dashboards for employees and senior management (Fig. S6, Supplementary Material 1). This enhanced data-driven decision-making and automated time-consuming reports.

The BI platform provides dashboards and a range of reports, such as an overall scorecard (weekly and month-to-date), top customer behaviour, account management report, top customer purchasing trends, customer analytics, and gap analysis (product) for each customer representative. The data and insights in each report are based on data collection and analysis carried out during CRM. Users can view information on all customer representatives or on a specific one, filtered by year and month. Within each dashboard, users can select the KPIs of interest. For example, in the top customer purchasing trend report, the KPIs include target sales versus actual sales, total revenue by sector, and top customers by purchasing amount (this year versus last year). In the scorecard report, KPIs include target versus actual sales and revenue (% to target by month), monthly sales rank (top customers), and the top 10 products sold (by sales value).

Following the design and development, the BI platform was implemented within SDG. Senior managers and employees received training on using the platform for decision-making. We developed a ‘Guidebook’ to help end users navigate the platform effectively. A video tutorial to guide staff in navigating the platform was also developed and made available to end users. We also ran a series of workshops to promote the platform and provide training, including platform usage, data governance, guidance on using the data through a new meeting format, an action-planning toolkit, and definitions and guidance on KPIs. The CRM-BI dashboard also facilitated weekly KPI scorecard meetings, which were attended by the business’s senior management. These meetings enhanced performance management within the business and increased levels of accountability. They also provided a forum for discussion and for ideas to improve KPI performance.

The platform fundamentally informed SDG’s approach to marketing, sales, and strategic planning, making the business more data-driven and analytical, and increasing visibility into performance and accountability within the organisation. The BI platform also allows SDG to fully capture the value of the data from its existing information systems. All senior managers in SDG have adopted the platform for data-driven planning and are increasingly making decisions based on objective evaluation and evidence.

## Evaluation

The overall CRM-BI solution, incorporating the strategic, operational, and technical artefacts, was evaluated using a summative ex-post evaluation through the final project evaluation reporting. This process involved completing a final project evaluation report, with questions answered separately by the associate, business, and academic teams. This ex-post evaluation focused on a retrospective assessment of the overall project and on predicting future benefits. In this section, we draw primarily on this evidence to evaluate the artefacts and the overall solution. Although this provides a reliable summative evaluation of the project, ongoing evaluation of artefacts through LMC meetings, fortnightly supervision meetings with the associate, and informal, ad hoc interactions between the associate and the company also informed the design of the artefacts and served as more informal formative evaluations.

Overall, the final evaluation report indicates that the project artefacts have been successfully embedded into the organisation. Feedback included in the final evaluation report from SDG’s chief executive indicates that the KTP project has delivered substantial benefits across multiple areas of the business. Based on the final project evaluation, SDG identified substantial business changes resulting from the project, including adopting strategic planning aligned with the strategic pillars and moving toward a data-driven culture in which all senior managers use data-driven approaches to planning, execution, and evaluation. The latter cultural change was facilitated by the BI platform, alongside training and workshops provided by the associate to company staff, and, more broadly, by the development of the organisation’s BI capability. In total, fifteen SDG staff were trained in the use of management dashboards, KPI-setting, value proposition, and account management. A permanent ‘Business Intelligence Analytics Manager’ position was also created as a direct result of the project. As a result of the project, additional staff are also being recruited in account management and business development.

The final report highlights the benefits of the project’s focal technical artefact, the CRM-BI platform, particularly the CRM-BI dashboard, which provides information to enhance decision-making. The CRM-BI platform enabled SDG to use data from its internal systems more effectively by providing staff with real-time analytics, advanced reporting, and market insights, thereby streamlining decision-making and improving strategic planning. Concrete examples of this include using the CRM-BI dashboard during meetings to drive discussion and decision-making, as well as a reported 25% reduction in the time the senior management team requires to prepare for meetings. This efficiency, coupled with time savings in automating reports, freed up time and resources, enabling staff to focus on more value-added activities. The CRM-BI platform also resulted in efficiency and time saving in the preparation of reports. In the final report, the business indicates that automating reports saved five senior team days per month. The new CRM-BI solution also provided real-time information, rather than the previous weekly and monthly reports. This provided more up-to-date information for enhanced decision-making. The associate also highlighted in the final report that data-driven decision-making has become routine in the business, with positive feedback and buy-in from stakeholders.

The project also resulted in improved data quality, which has positively impacted planning and tracking capabilities, helping alleviate downward margin pressures. For example, the war in Ukraine resulted in direct and indirect increases of up to 35% in product costs. Operating costs have also risen by up to 25%. With the aid of the BI platform, SDG has improved margin verification, sales planning, and pricing operations.

Improving relationship marketing also created significant value for SDG. The customer engagement model enabled SDG to prioritise key accounts, enhance customer interactions, and identify growth opportunities. The model provides enhanced visibility of performance metrics across the business, ensuring decision-makers have the insights required for effective decision-making. The introduction of CRM further strengthened SDG’s market position by prioritising key relationships and delivering a more tailored customer experience.

The project also delivered measurable financial benefits. During the project, annual sales turnover increased by £100,000, with a three-year forecast projecting growth to £1.5 million. Current export revenue has grown by £250,000 as a result of the project, with a three-year projection of an additional £15 million in export revenue resulting from the project. This is attributed to improved business intelligence, market lead qualification and improved price setting. Annual profit before tax also saw a measurable increase, with current annual profit before tax rising by £200,000, directly attributable to the project. The predicted three-year increase in profit before tax resulting from the project is £500,000. This includes both new products and new markets, as well as operational efficiency and improved quality. These performance gains are attributed to improved account management, margin planning, optimising pricing, predictive stock planning and sales, and improved key account reporting. SDG has also strengthened its competitive position, delivering enhanced customer value and building long-term resilience. Ultimately, the project has transformed SDG into a more innovative, customer-focused, and data-driven organisation.

Although the overall evaluation of the solution was largely positive, the associate also highlighted areas for further work in the final report, including expanding the business’s BI and analytics staff, formalising data governance and standard operating procedures, and continuing to provide training and workshops to strengthen data-driven decision-making across the organisation.

## Design principles

Based on the design and implementation of the CRM-BI platform and associated strategic and operational artefacts, we propose design principles to guide the development of CRM-BI solutions in similar contexts. These design principles emerged from the design process and are embodied in the overall solution.

The first design principle focuses on the overall relationship and sequencing of the full suite of organisational and technical artefacts that make up the solution. The overall solution highlights the need for the customer strategy, key account relationship model, and service blueprint to precede the development of the CRM-BI technical solution. This is because these organisational CRM artefacts are necessary to inform the design of the CRM-BI platform. To successfully develop the customer strategy also requires analysing data on current customers, including segmentation and value analysis. This initial analysis is necessary to develop the strategy, which subsequently informs the CRM-BI platform. This analysis can also help identify important KPIs and issues with data access and quality, which are critical to the development of the CRM-BI platform.

For example, these organisational artefacts should be used to inform the data that is presented in the dashboard KPIs. This is necessary to ensure that the dashboard design aligns with the organisation’s strategic and operational objectives. Organisations have access to a vast array of data, making it necessary to focus on the most important KPIs and metrics. Failure to incorporate key insights from strategic and operational artefacts could result in a dashboard that presents irrelevant information, making it difficult for decision-makers to focus on what is most important to the organisation’s goals and vision.

DP1 Strategic and organisational CRM artefacts, including customer strategy and relationship management processes, should precede and inform the overall strategy and the design of the technical BI solution.

The business’s overarching strategy should also inform the design of the CRM-BI dashboard. The broader organisational strategy developed as part of the project is embodied in the organisation’s strategic pillars. These strategic pillars directly highlight the need for a business intelligence solution, specifically a CRM-focused one. The business’s core strategic objectives of smart growth, innovation and customer centricity all require data and insights, with smart growth and customer centricity requiring CRM data and insights. The business’s innovation objectives centre on making more effective use of data to support other strategic objectives. To ensure the project’s overall success, the technical BI platform artefact was driven by these strategic objectives, rather than pursuing analytics for its own sake without a clear business objective. The strategic pillars also informed the type of data and analytics required, which, in this project, focused on CRM. The detailed KPIs presented in the dashboard, therefore, centre on areas of strategic importance and are also informed by the CRM artefacts. Given the need to ensure that BI is driven by strategic objectives, we propose this as a core design principle.

DP2 CRM-BI design and instantiation should be driven by the organisation’s strategic objectives.

Our overall solution design highlights the need to combine both qualitative and quantitative information to develop the CRM-BI solution. For example, to effectively manage key accounts, quantitative data on orders and financial performance were required, alongside more qualitative information on key members of the customer organisation, their priorities, and the quality and strength of the relationship. Combining qualitative and quantitative insights in the key account relationship artefact ultimately yielded a more effective solution than relying on either approach in isolation.

DP3 Combine quantitative customer analytics with qualitative relationship mapping.

To develop the customer engagement model, it was necessary to map the customer journey stages and capture data related to each stage. Much of the data required at this stage was not held in internal information systems, necessitating collection from a range of sources, including websites, social media, emails, satisfaction scores, and qualitative feedback. The specific data required and its sources differed across the customer journey. To effectively capture the required data, it was therefore necessary to map stages of the customer journey to data sources. The collection and analysis of this data provided crucial insights into business opportunities at each stage of the customer journey, which informed the customer engagement model. For example, business insights included development opportunities, potential leads, and referrals.

DP4 Link customer journey stages to specific data-capture and insight-generation mechanisms.

To ensure the successful rollout and use of BI dashboards, they need to be embedded in wider organisational processes, including governance, training and meeting routines. From a governance perspective, two areas were particularly important to SDG. The first was ensuring data security and privacy, achieved through secure data storage, role-based access, and dashboard views. This meant that end users could only view appropriate data. The second was to ensure appropriate processes and policies for the collection, storage, and use of data within the organisation. This helped formalise these processes and improve data quality.

End users were also trained to use the dashboard’s functionality and interpret the data. As analytics capability was new to SDG, upskilling staff was crucial to ensure that analytics led to improved decision-making. The dashboard also became a central part of meetings within the organisation, becoming embedded in the typical meeting routine. This helped to ensure that decisions within the organisation became more data-driven.

DP5 Embed dashboard deployment in governance, training, and meeting routines.

Our final design principle focuses on the overall process and the roles of the associate, business, and academic teams in the project. The associate developed the project’s main artefacts, with support from the academic team and the business. Crucial to this process was close communication among the associate, business, and academic teams. The associate was embedded within the business throughout the two-year project, facilitating ongoing communication and refinement of artefacts and incorporating qualitative business insights and knowledge into artefact design. The associate also maintained close communication with the academic team through fortnightly supervision meetings, facilitating knowledge transfer and further enhancing the design of the artefacts. This interaction was crucial to the design of the artefacts and represented a key process-level design principle. This close interaction also helped ensure the artefacts were grounded in business knowledge and met the organisation’s requirements. Ultimately, the CRM-BI solution designed in this project would have been more challenging to design and implement through an arm’s-length consultancy relationship.

DP6 The developer of CRM-BI solution artefacts should be embedded within the organisation, with close communication among stakeholders to ensure that business and academic knowledge is embedded in the artefacts.

## Discussion

Following the design science methodology, we undertook a collaborative university-industry project with an SME supplier in the construction industry to design, develop and implement a CRM-BI solution within the business. The design and implementation of the CRM-BI solution have resulted in a range of benefits for the business. The project enables employees and the senior management team to take a more data-driven approach to CRM decision-making. The CRM artefacts provide a framework for guiding customer interactions and measuring performance. The design and implementation of the CRM-BI solution and the associated training also helped drive cultural change within the business, leading to new CRM processes, continuous improvement, and increased accountability. Increased automation of data analysis has resulted in time savings and greater efficiency. From a practical perspective, the benefits of the project helped the company achieve its overarching business aim of improving CRM and ultimately business growth.

From a research perspective, we contribute new design knowledge. Our main proposition from the study is that integrating the design of CRM and BI artefacts is more effective than designing these artefacts in isolation. This proposition is captured in our overarching framework and design principles. The guiding framework links the key artefacts in developing the CRM-BI solution. This framework builds on the CRM literature, which conceptualises CRM as comprising technologies, business processes, and strategies (Zablah et al., 2004). We build on these multidimensional and interrelated perspectives on CRM, presenting an integrated framework that links strategic, process, and technical artefacts. Our framework provides more depth than existing conceptualisations by presenting the specific artefacts within these categories as well as their interlinkages. Although there has been debate in the design science literature about whether organisational artefacts are appropriate in design science research (Winter, 2008), we view these as crucial components of our overall information system alongside the focal technical artefact. This approach aligns with the recognition in the broader information systems literature that both technical and organisational components are important (Winter, 2008).

The study resulted in design principles that can be considered when developing CRM-BI solutions in SMEs. Our artefacts and design principles include the CRM-BI platform as the central technical artefact, and demonstrate how this is underpinned by strategic and business process artefacts. These design principles support existing research highlighting the importance of data governance (Corallo et al., 2023), data quality (Corallo et al., 2023; Hausladen & Schosser, 2020), training, skills and leadership (Cosic et al., 2015; Klievink et al., 2017), and technical resources (Comuzzi & Patel, 2016; Gökalp et al., 2021). However, we also extend this literature, highlighting the interrelationships between strategic, process, and technical artefacts in developing solutions.

The design principles from our integrated CRM-BI solution point to the need to draw on strategic and process artefacts to inform the development of the technical solution. Studies have indicated that decision-makers can typically only hold a small amount of information in short-term memory, often posited as around 3–5 chunks of information (Cowan, 2001). This means that it is necessary to carefully design dashboards that present only the most important information. Incorporating irrelevant and unnecessary information can result in ‘paralysis by analysis’ (Langley, 1995). Drawing on strategic and operational artefacts in dashboard design, alongside ongoing engagement with end users, can help ensure that dashboards are effective tools for decision-making.

During the project, we encountered several challenges in developing the CRM-BI platform. Here, we discuss our approach to overcoming them, with the aim of providing further insights to guide the management of CRM-BI projects. The first and most critical one is the availability and ease of access to existing data and the lack of a clear, concise data governance structure. The data is stored in different units or functions across SDG and is also held by external system suppliers. We had to develop a deep understanding of the business processes and practices to obtain relevant information. Usage of third-party applications to collect and store customer/product data made it challenging for SDG to access its own up-to-date data. The second is the organisational challenge, which underlines the lack of analytical competency in the existing workforce and the struggle to build a data culture among employees. Because employees lack the knowledge and capability to conduct in-depth data analysis, securing their buy-in for data-driven strategy planning is challenging. Third, scaling and automating data analysis and reporting is another challenge, given the company’s heavy reliance on external parties to generate data-driven reports. The fourth challenge is change management. The team invested substantial effort into building trust with employees to promote the changes in the business. This focused on gathering employee opinions to inform the design and on creating a collective effort, ultimately leading to buy-in for the changes.

The final project evaluation highlighted two aspects essential to further promote the adoption of business analytics to drive growth beyond the project. The first is maintaining momentum. Ownership and accountability for data-driven decision-making are critical among staff. The senior management team led by example during the platform’s implementation. SDG has devised systems and processes to implement the new practices, and the senior management team have worked to integrate these into employees’ daily routines alongside allocating responsibility for KPIs. Focusing on BI development should be viewed as an ongoing function within the company. The second is employee upskilling. The project highlights the importance of developing internal analytics human capital to enable the data analysis, interpretation of insights, and implementation of findings. This capability development requires ongoing training to develop initial capabilities as well as developing capabilities in emerging analytics technologies. Recruitment of external analytics staff, alongside internal capability building, also helped to strengthen the business’s overall CRM-BI capability.

In addition to its practical contributions, the study advances the business analytics literature by integrating CRM and BI. Existing design science literature focusing on business analytics has drawn on kernel theory from areas such as criminology (Vо & Plachkinovа, 2023), socio-technical systems theory (Dremel et al., 2020), and the theory of effective use (Ruoff et al., 2023). Our findings contribute to this literature by incorporating CRM alongside BI into the design, development, implementation, and evaluation of the CRM-BI platform artefact and the supporting strategic and process artefacts. This approach has revealed specific benefits, challenges, and opportunities from CRM-BI solutions. Businesses can draw on these insights to innovate with and enhance their CRM-BI capabilities, with the aim of enhancing firm performance.

## Conclusions

Designing and implementing CRM-BI solutions is challenging for SMEs, involving the creation of strategic, process, and technical artefacts. Drawing on the design science method to address our initial research questions, this study provides a rich description of the design of CRM-BI artefacts in an SME, as well as new design knowledge in the form of design principles. The findings highlight the interlinkages between artefacts and the need for a holistic approach to developing CRM-BI capabilities in SMEs.

### Limitations and future research

As with other design science research, the main limitation concerns the generalisability of the findings to other contexts. Although we ground our design science approach in CRM and BI theory and practice, it is important to keep in mind that the study’s empirical component is situated within a specific firm and industry, focusing on a single case. For example, artefacts such as the cluster analysis are based on firm-specific data. The specific strategies and processes designed should also be generalised with caution, considering differences in context. Future studies could consider CRM-BI applications in other contexts to explore the generalisability of the findings to other industries. Both CRM and BI are important across all industries, creating the potential to apply the design science methodology to address similar organisational problems in diverse contexts. Future work could also extend the framework to other functions of the business, for example, a similar approach could be adopted in areas such as finance or human resource management, combining the strategic and organisational artefacts with BI platform design. This could help to establish the generalisability of the findings to other business functions. Although we ground the project in established theory, it is difficult to establish causality. Future work could therefore draw on the framework presented in this study to examine causality using other methods, such as experimental approaches.

Our design and evaluation approach also has certain limitations arising from the nature and design of the overall project. Rather than proceeding through clearly delineated design cycles, the project was carried out based on a workplan. In the context of the SME, formative feedback was often ad hoc and informal, with issues and updates to artefacts occurring in a less-structured fashion. This may be partly due to the SME context of the project, and partly due to following an existing KTP workplan-based approach. Our overall artefact evaluation approach also focuses on summative evidence documented in the final report, with questions completed independently by the company, academic team, and associate. Although this provides triangulation from multiple sources, other evidence would also be useful, such as ongoing survey data from end users and, potentially, experimental evidence demonstrating the impact of the artefacts. The completion of the final report and evaluation was carried out by project participants, introducing potential bias into more qualitative assessments of the project.

Although our project lasted for two years, future work could consider extending the evaluation period to account for longer-term benefits from the artefacts, as well as identifying any longer-term risks and issues that may arise. This longer-term evaluation may be particularly useful in the context of rapidly evolving areas of CRM and BI. In this context, it is likely that the original design requirements are updated and change to meet the evolving business needs, as well as to take advantage of new technological developments.

Future work could also consider extending the technical BI solution. This could include incorporating predictive and prescriptive analytics. Future solutions could also draw on generative AI in dashboard design to enable more interactive and conversational interfaces. This could help make the BI platform more accessible to staff who lack the technical skills to interact with the graphical user interface. Generative AI could also be used in future studies to assist in the design of strategic and organisational artefacts, such as planning, writing, and editing strategic CRM documents.

## Supplementary Information

Below is the link to the electronic supplementary material.

Supplementary Material 1

## Data Availability

No datasets were generated or analysed during the current study.

## Footnotes

1 https://wearesdg.com/.

2 https://iuk-ktp.org.uk/case-study/harnessing-the-power-of-data-and-automation.

3 https://www.ukri.org/councils/innovate-uk/.
