# Supplementary material for: Integrating customer relationship management with business intelligence platform design: a design science approach
Source: J Digit Manag. 2026 Sep 15;2(1):3. doi: 10.1007/s44362-026-00024-x (PMC13602855; doi:10.1007/s44362-026-00024-x)
Supplement: Supplementary file 1 [file 44362_2026_24_MOESM1_ESM.docx]

**Table S1.** Selected studies on the resources for applying data analytics

| **Study** | **Resources for applying data analytics** |
| --- | --- |
| Cosic et al. (2015) | Governance, culture, people, and technology |
| Comuzzi & Patel (2016) | Strategic alignment, organisation, data, and information technology |
| Klievink et al. (2017) | Organisational alignment, organisational maturity, and organisational capabilities for big data use |
| Lismont et al. (2017) | Data, enterprise or organisation, leadership, targets or techniques, applications and analysts |
| Hausladen & Schosser (2020) | Strategic alignment, organisation, data, information technology |
| Gökalp et al. (2021) | Organisation, strategy management, data analytics, data governance, technology management, supporting |
| Corallo et al. (2023) | Organisation, data management, data analytics, infrastructure, governance and security |
| Menukin et al. (2023) | Organisation, information technology and analytics infrastructure, analytics processes, skills, governance, data and analytics technologies |

**Figure S1.** An example of customer analytics (MC)

1. Customer buying behaviour

MC decreased sales in 2021 and displayed a YoYo effect in the trend. The goal is to find out why the sales are declining and try to increase them again.

Are these declines due to?

- Weak customer relationship
- Multiple customer accounts
- Customer project-related work
- Customer is doing less business

2. Customer lifetime value (CLV)

What does this mean?

- The total customer lifetime value for MC is £292,412 from 2018 to 2021, which means to maintain this rank/position, there needs to be sales of at least £73,103, which is not the case.
- The goal is to increase sales to at least £100,000, which will raise the rank and ultimately increase the customer's overall lifetime value.

3. Product purchase trend

- 30 Products consistently purchased year-on-year: MC purchased 74 products in 2021. Of these, 30 products (40%) were consistently purchased each year from 2018 to 2021. MC is likely to purchase these 30 products in 2022 as well.
- 81% of predictive products: There is a high probability of purchasing 30 further products, enabling 81% of predictive products. We know what they are likely to buy; we just need to push them more.
- Limited trend for 123 products: The remaining 123 products show limited trends. Can we conduct further research to determine whether these products were one-time purchases for project-related work, or whether they purchase from us only when they have no other option?

**Note:** The customer’s name has been replaced with a descriptive label (MC). The numbers have been processed and are for illustrative purposes only.

**Table S2:** Detailed description of each cluster

| **Clusters** | **True Blues** | **Safeguard Strategic** | **Attention Seekers** | **Passive clients** |
| --- | --- | --- | --- | --- |
| **No. of Customers** | 31 | 102 | 29 | 144 |
| **Years in business:** | Min: 3 years  Max: 76 years  Mean: 37 years | Min: 1 years  Max: 150 years  Mean: 33 years | Min: 3 years  Max: 70 years  Mean: 22 years | Min: 6 years  Max: 110 years  Mean: 23 years |
| **Years with SDG:** | Min: 2  Max: 30 years  Mean: 15 years | Min: 0  Max: 23 years  Mean: 9 years | Min: 1  Max: 23 years  Mean: 10 years | Min: 0  Max: 23 years  Mean - 9 years |
| **Company turnover:** | Min: £1m  Max: £800m,  Mean: £120m | Min: £3m  Max: £1billion,  Mean: £90m | Min: £1.1m  Max: £45m  Mean: £10.77m | Min: £268,399  Max: £19m  Mean: £8.25m |
| **Average yearly revenue** | Min:£3,260  Max: £852,878  Mean: £133,948 | Min: £0  Max: £18,305  Mean: £2784 | Min: -£3195  Max: £80,447  Mean: £17,483 | Min: -£1689  Max: £14,155  Mean: £1, 898 |
| **Average yearly order frequency** | 986 | 52 | 189 | 54 |
| **Customer Lifetime Value** | Min: £48,955  Max: £3.65m  Mean £573,238 | Min: £0  Max: £87,517  Mean £14,843 | Min: £3221  Max: £375,339  Mean £72,885 | Min: £0  Max: £56,146  Mean - £10,267 |
| **Total yearly revenue** | £3m | £281,202 | £506,993 | £273,272 |
| **Total CLV from all these customers** | £13.18m | £1.52m | £2.11m | £1.47m |

**Table S3:** Detailed customer strategies for each customer segment

| **Customer segment** | **Relationship strength** | **Customer strategy** |
| --- | --- | --- |
| True-blues:  Manage closely | Supporters | - Keep them in the loop with regular product updates, follow-ups and informal contact. - Engage with them more through informal contact (e.g., go out, entertainment) or in-person catch-ups. - Effective account management: service management, stock management, and all staff must be up to date with the stock and pricing. - Keep contacts happy, try to build as many relationships as possible. - Online training, in-house CPD (continuing professional development). - Regular communication to check if they are happy. - Regular communication to discuss pricing. |
|  | Neutrals | - Formal updates may suffice; however, is there anything that might help garner support? - Understanding their business, different conversations around what SDG can offer them. - Occasional informal contact to build their trust, being available for them, effective pricing strategies, and project-specific communications. - Focusing on marketing actions to communicate how SDG can support projects. - Shout out on social media, ensure the customer feels valued. - Online training, in-house CPD. |
|  | Defectors | - Ensure that they know that their concerns are recognised and dealt with effectively. - Try to communicate with them and gauge their satisfaction through feedback/surveys. - Understanding their business, research the customers to gather more information on them before reaching out. - Know what project they are working on, what they buy and when they buy it. - Building the relationship by picking the key contacts within the organisation. - Educating them on what we can offer them, and providing technical advice wherever needed. - Marketing actions: show them that we care. |
| Safeguard strategic: Keep satisfied | Supporters | - Use concise updates to inform of new products/ relevant services SDG offers. - Add specific informal contact as necessary. - Build relationships to improve customer engagement and activity. |
|  | Neutrals | - Keep informed and be prepared to field queries as they arise. - Ensure concerns are addressed, including follow-up to gain feedback. - Address any complaints promptly. - Gain an understanding of the customer and how SDG can provide support. - Identify any pain-points and resolutions. - Provide opportunities for entertainment and team-building. |
|  | Defectors | - Ensure that specific concerns are responded to promptly. - Consider a pricing change for these customers to win them back or avoid letting them go to competitors. - Have more interaction with the buyers and have answers to their questions. - Ensure effective account management and risk management. - Strategic targeted sales/marketing more in terms of technical proposals and understanding, making them more aware that SDG is there to help them and solve their problem. - Ensure effective communication with customers at a strategic level to identify areas where SDG can provide support. |
| Attention seekers:  Keep informed | Supporters | - Identify key motivations for customers in this category. - Identify opportunities to support the customer. - Maximise opportunities for bulk orders to increase efficiency, whilst considering pricing and transportation costs. - Establish relationships with customers to facilitate direct communication. - Ensure effective account management and time management. |
|  | Neutrals | - Identify opportunities to win customers over. - Identify opportunities to convert these customers to supporters. - Ensure effective relationship management by geographic area. - Educate customers on new products and alternative options or solutions. - Ensure effective marketing of the SDG brand. |
|  | Defectors | - Identify the best way to reach these customers. - Identify opportunities to address concerns and gain support. - Provide defectors with information on SDG’s service and values. - Ensure pricing is appropriate. |
| Passive clients:  Minimum Effort/ Monitor | Supporters | - Provide general marketing communications. - Supplement with specific contact as needed, whilst maintaining efficiency. - Ensure account management is responsive. - Carry out an annual review to evaluate customers' needs and any changes to their business. |
|  | Neutrals | - General communications can be supplemented with a specific contact if necessary. - Carry out an annual review to evaluate customers' needs and any changes to their business. - Provide one-to-one interaction as necessary. |
|  | Defectors | - Allay any concerns via general channels. - Maximise efficiency by providing general communications rather than one-to-one interaction. - Responsive contact purely for internal sales. |

**Figure S2.** Templates for key account management

1. Template to capture customer data

2. Templates for relationship mapping

**Note:** The information (e.g., email and phone number) shown has been processed for illustrative purposes only.

**Figure S3.** Framework for capturing customer insights along the customer journey

**Figure S4.** Customer survey

**Figure S5.** Customer feedback

**Figure S6.** An illustration of the business intelligence platform
